# Uncoupling neuronal death and dysfunction in *Drosophila* models of neurodegenerative disease

**DOI:** 10.1186/s40478-016-0333-4

**Published:** 2016-06-23

**Authors:** Amit K. Chouhan, Caiwei Guo, Yi-Chen Hsieh, Hui Ye, Mumine Senturk, Zhongyuan Zuo, Yarong Li, Shreyasi Chatterjee, Juan Botas, George R. Jackson, Hugo J. Bellen, Joshua M. Shulman

**Affiliations:** Department of Neurology, Baylor College of Medicine, Houston, TX 77030 USA; Department of Neuroscience, Baylor College of Medicine, Houston, TX 77030 USA; Department of Molecular and Human Genetics, Baylor College of Medicine, Houston, TX 77030 USA; Program in Developmental Biology, Baylor College of Medicine, Houston, TX 77030 USA; Department of Neurology, University of Texas Medical Branch, Galveston, TX 77555 USA; Parkinson’s Disease Research, Education, and Clinical Center, Michael E. DeBakey VA Medical Center, Houston, TX 77030 USA; Howard Hughes Medical Institute, Baylor College of Medicine, Houston, TX 77030 USA; Jan and Dan Duncan Neurological Research Institute, Texas Children’s Hospital, 1250 Moursund Street, Suite N.1150, Houston, TX 77030 USA

**Keywords:** Alzheimer disease, Parkinson disease, MAPT, Tau, Amyloid-beta peptide, SNCA, alpha-synuclein, Neurodegeneration, Neurophysiology, Synapses, Animal model, Drosophila

## Abstract

Common neurodegenerative proteinopathies, such as Alzheimer’s disease (AD) and Parkinson’s disease (PD), are characterized by the misfolding and aggregation of toxic protein species, including the amyloid beta (Aß) peptide, microtubule-associated protein Tau (Tau), and alpha-synuclein (αSyn) protein. These factors also show toxicity in *Drosophila*; however, potential limitations of prior studies include poor discrimination between effects on the adult versus developing nervous system and neuronal versus glial cell types. In addition, variable expression paradigms and outcomes hinder systematic comparison of toxicity profiles. Using standardized conditions and medium-throughput assays, we express human Tau, Aß or αSyn selectively in neurons of the adult *Drosophila* retina and monitor age-dependent changes in both structure and function, based on tissue histology and recordings of the electroretinogram (ERG), respectively. We find that each protein causes a unique profile of neurodegenerative pathology, demonstrating distinct and separable impacts on neuronal death and dysfunction. Strikingly, expression of Tau leads to progressive loss of ERG responses whereas retinal architecture and neuronal numbers are largely preserved. By contrast, Aß induces modest, age-dependent neuronal loss without degrading the retinal ERG. αSyn expression, using a codon-optimized transgene, is characterized by marked retinal vacuolar change, progressive photoreceptor cell death, and delayed-onset but modest ERG changes. Lastly, to address potential mechanisms, we perform transmission electron microscopy (TEM) to reveal potential degenerative changes at the ultrastructural level. Surprisingly, Tau and αSyn each cause prominent but distinct synaptotoxic profiles, including disorganization or enlargement of photoreceptor terminals, respectively. Our findings highlight variable and dynamic properties of neurodegeneration triggered by these disease-relevant proteins in vivo, and suggest that *Drosophila* may be useful for revealing determinants of neuronal dysfunction that precede cell loss, including synaptic changes, in the adult nervous system.

**Electronic supplementary material:**

The online version of this article (doi:10.1186/s40478-016-0333-4) contains supplementary material, which is available to authorized users.

## Introduction

Alzheimer’s disease (AD) [46] and Parkinson’s disease (PD) [32] are common and incurable neurodegenerative disorders responsible for substantial cognitive and/or motor disability in the aging population. Both disorders are characterized at autopsy by widespread neuronal loss within the central nervous system in association with aggregated and misfolded toxic protein species. AD is neuropathologically defined by the presence of amyloid plaques and neurofibrillary tangles, predominantly consisting of extracellular Aß peptide deposits and intracellular filamentous aggregates of the microtubule associated protein Tau (MAPT/Tau), respectively. Similarly, intracellular inclusions of alpha-synuclein protein (SNCA/αSyn) comprise the Lewy bodies that pathologically define PD. Importantly, since rare mutations in the *amyloid precursor protein*, *MAPT*, and *SNCA* genes all cause Mendelian, young-onset forms of dementia and/or parkinsonism, the misfolded and aggregated proteins that form pathological inclusions in AD and PD are causatively linked to disease pathogenesis. Further support comes from numerous transgenic animal models [16, 23] in which expression of Aß, Tau, or αSyn have been demonstrated to recapitulate neurotoxicity and often other clinicopathologic features relevant to human disease.

AD and PD are also similarly characterized by a prolonged, insidious onset in which absent or subtle symptoms precede more severe cognitive and motor disability and clinical recognition. While part of the age-dependent progression certainly relates to the cumulative toll of neuronal loss and failure of compensatory mechanisms, it is increasingly apparent that a period of cellular dysfunction precedes death [27]. For example, fibrillar Aß pathology has been associated with altered measures of brain metabolism [3, 31] and connectivity [55] and these changes might potentially be explained in part by synaptic loss and dysfunction seen in experimental models. Similarly, clinicopathologic studies of PD suggest dopaminergic terminal dysfunction precedes nigral cell death [29], potentially contributing to early clinical manifestations of disease. Indeed, a current priority is to develop strategies to identify affected individuals earlier allowing potential therapeutic intervention before irreversible cell death [54].

Robust experimental models of neuronal dysfunction in AD and PD are therefore urgently needed, including systems amenable to rapid genetic manipulation and medium to high-throughput screening. The fruit fly, *Drosophila melanogaster*, has been a valuable tool for studying neurodegenerative disorders, and numerous transgenic models have been reported based on expression of human Aß, Tau, and αSyn. In particular, given its accessibility and dispensability, the fly eye has proven a versatile experimental system, and Aß, Tau, and αSyn have all been associated with retinal toxicity [9, 20, 28, 33, 52]. However, prior studies of these *Drosophila* AD/PD transgenic models have used different expression conditions and assays, hindering systematic comparisons of toxicity profiles. Further, many studies have utilized expression drivers that do not allow differentiation of toxicity in (1) the adult versus developing nervous system and/or (2) neuronal versus non-neuronal supporting cells (glia). Here, we use standardized conditions and assays to systematically examine neurodegenerative changes induced by Aß, Tau, and αSyn, including the evaluation of both structural and functional changes following selective expression in the neuronal cells of the adult retina. These studies demonstrate that each protein recapitulates a unique profile of neurodegenerative pathology, demonstrating distinct and apparently separable impacts on neuronal death and dysfunction.

## Materials and Methods

### *Drosophila* Stocks and husbandry

The following previously reported *Drosophila* stocks were obtained for our experiments: *Rh1-GAL4 (II)*[63], *UAS-Tau*^*WT11*^ [62], and *UAS-alpha-synuclein* [18]. The *UAS-Tau*^*WT11*^ transgene expresses the full-length, 383-amino acid isoform of human Tau, lacking amino terminal inserts (exons 2 and 3) and containing 4 microtubule binding repeats (0N4R). The *UAS-Aß* and codon-optimized *UAS-αSyn* strains are newly reported in this study but are both available in the Bloomington *Drosophila* Stock Center: *P{UAS-APP.Abeta42.B}m26a* and *P{UAS-SNCA.J}7*. The *UAS-Aß* line consists of the open reading frame encoding the 42 amino acid Aß fragment of human *APP* fused with the fly *argos* secretion signal peptide under the control of UAS in the P{UAST} vector. The resulting amino acid sequence is (*argos* sequence italicized): *MPTTLMLLPCMLLLLLTAAAVAVGG*DAEFRHDSGYEVHHQKLVFFAEDVGSNKGAIIGLMVGGVVIA

Expression of the Aß peptide was confirmed and quantified by ELISA (Additional file 1: Figure S1). To extract total Aβ42, 7 heads were homogenized in 50 μL GnHCl extraction buffer (5 M guanidinium HCl, protease inhibitor cocktail (GenDEPOT) and 50 mM Tris HCl, pH = 8.0). Following centrifugation (14,000 g for 15 min @ 4 °C), supernatant was diluted 1:50, 1:100 and 1:200 in standard dilution buffer, before performing ELISA measurements (Invitrogen, KHB3441) following the manufacturer’s protocol. ‬ The codon-optimized *UAS-alpha-synuclein* line was developed from a synthetic human *alpha-synuclein* cDNA engineered by DNA 2.0 (Menlo Park, CA) using codons optimized for insect expression. The resulting cDNA, encoding full-length, wild-type human alpha-synuclein protein, was subcloned into the pExpress-UAS expression vector [43]. Expression of αSyn was confirmed by immunoblot (clone 42, BD Transduction Laboratories, 1:1000) (Additional file 1: Figure S1). Immunoblot was also performed for Tau (Dako, 1:5000). Expression of Tau and αSyn were quantified relative to recombinant, purified protein standards of known concentration (Tau: Abcam, Cambridge, UK; αSyn: EMD Millipore, Temecula, CA). All crosses were established at 18 °C, and adult progeny were transferred to 25 °C between 0–24 hours post-eclosion for aging and subsequent analyses. Flies were maintained in continuous, ambient laboratory light conditions. Female flies were used for all analyses.‬‬‬‬

### Retinal histology

Adult flies were fixed in 8 % glutaraldehyde (EM grade) and embedded in paraffin. Frontal (5 μm) and tangential (3 μm) retinal sections were prepared on the microtome (Leica) and stained with hematoxylin and eosin. At least 4 animals were examined for each genotype and preparation (frontal and tangential sections). Frontal retinal sections (Fig. 2) were photographed at the level of the giant commissure interneurons, posterior to the fan-shaped body. As in prior work [40], we quantified the number of ommatidia in which 7 photoreceptor rhabdomeres remained intact within each field of view (100x) centered within well-oriented, retinal sections from each animal (Fig. 3).

### Electrophysiology

ERG recordings were performed as previously described [64]. In brief, adult flies were anesthetized and glued to a glass slide. A reference electrode was inserted in the thorax and the recording electrode was placed on the corneal surface of the eye. Flies were maintained in the darkness for at least 5 minute prior to a train of 10, 1-second flashes of white light pulses (LED source with daylight filter), during which retinal responses were recorded and analyzed using WinWCP (University of Strathclyde, Glasgow, Scotland) and AxoGraph (Berkeley, CA), respectively. At least 6 flies were examined for each genotype and timepoint.

### TEM

Both retinae and laminae in adult flies were processed for TEM imaging as described [58]. Samples were processed using a Ted Pella Bio Wave microwave oven with vacuum attachment. Adult fly heads were dissected at 25 °C in 4 % paraformaldehyde, 2 % glutaraldehyde, and 0.1 M sodium cacodylate (pH 7.2). Samples were subsequently fixed at 4 °C for 48 hours. 1 % osmium tetroxide was used for secondary fixation and subsequently dehydrated in ethanol and propylene oxide, and then embedded in Embed-812 resin (Electron Microscopy Science, Hatfield, PA). 50 nm ultra-thin sections were obtained with a *Leica UC7* microtome and collected on Formvar-coated copper grids (Electron Microscopy Science, Hatfield, PA). Specimens were stained with 1 % uranyl acetate and 2.5 % lead citrate and imaged using a JEOL JEM 1010 transmission electron microscope with an AMT XR-16 mid-mount 16 mega-pixel CCD camera. For quantification of ultrastructural features, electron micrographs were examined from 3 different animals per genotype.

### Statistical analysis

**Fig. 1:**
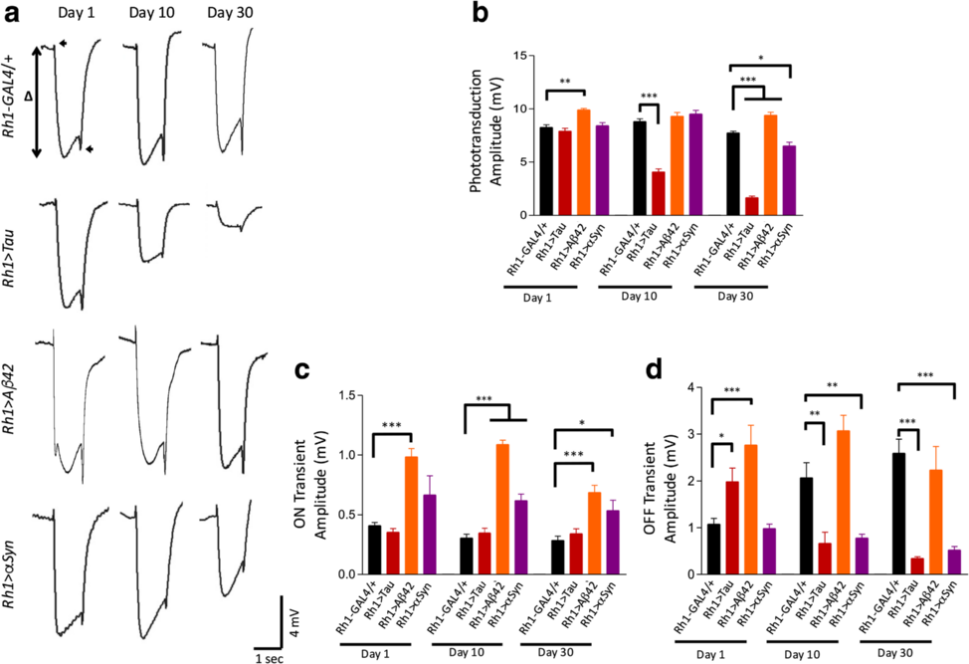
Age-dependent changes in retinal function monitored using electroretinograms. **a** Representative ERG traces are shown for each genotype examined at 1, 10, and 30 days. The overall depolarization amplitude (Δ) reflects the output of the phototransduction cascade. Synaptic neurotransmission generates the ON and OFF transient potentials (arrowheads). **b** Quantification of ERG depolarization amplitude by genotype and age (2-way ANOVA model F-test, p < 0.001). *Rh1 > Tau* causes a rapid and progressive loss of ERG depolarization whereas *Rh1 > αSyn* causes a relatively modest and late decline. **c & d** Quantitation of the synaptic ON and OFF transient potentials reveals efficacy of photoreceptor neurotransmission (2-way ANOVA p < 0.001 for each model). The measurement of the ON transient **c** is less reliable due to its small magnitude in controls (panel A). *Rh1 > Tau* and *Rh1 > αSyn* show progressive loss in OFF transients **d**. ERGs were recorded from at least 6 animals per genotype (genotype, n_1_, n_10_, n_30_): *Rh1-Gal4/+*, 17, 13, 14; *Rh1 > Tau*, 16, 16, 11; *Rh1 > Aß*, 6, 12, 6; *Rh1 > αSyn* 9, 10, 14. Subsetted t-tests were performed for post-hoc comparisons of control animals with each experimental genotype, considering each timepoint independently, and p-values were adjusted using the Bonferroni method. *, *p* < 0.05; **, *p* < 0.01; ***p < 0.001

For primary analyses of ERG (Fig. 1) and rhabdomere loss (Fig. 3), two-way ANOVA was implemented to detect potential differences between group mean outcomes by genotype and age. The overall model F-test is reported in the relevant figure legends. Subsequently, subsetted student’s t-tests (2-tailed) were performed for post-hoc comparisons of control animals with each experimental genotype (Tau, Aß, or αSyn), considering each timepoint independently; Bonferroni adjustment was performed in order to correct p-values for multiple comparisons [p_adjusted_ = p_unadjusted_ * 3, for n = 3 genotypes). For secondary analyses of TEM parameters, statistical comparisons were implemented using a two-tailed student’s t-test. Error bars in all analyses represent the standard error of the mean (SEM, 95 % confidence interval).

## Results

The transgenic models utilized for this comparative study take advantage of the GAL4-UAS system [7], in which cDNAs encoding human Tau (0N4R isoform), the Aß42 peptide, or full-length αSyn protein are under control of the heterologous yeast *Upstream Activating Sequence* (*UAS*) promoter element and responsive to driver lines expressing the GAL4 transcription factor. We selected the *Rhodopsin1-GAL4* (*Rh1*) line that is expressed following photoreceptor maturation and is thus only active following completion of eye development beginning in late pupal stages [47]. This permits the study of potential toxic properties within neurons, excluding the potential contribution of glial or supporting cells. In addition, use of the *Rh1* driver further excludes the potential delayed contribution of developmental toxic mechanisms. Since the GAL4-UAS system is temperature sensitive, all crosses were additionally carried out at 18 °C, and shifted to 25 °C following eclosion, further restricting transgene expression to adulthood [39]. In order to facilitate comparisons of resulting toxicities, we determined the absolute expression level of Tau, Aß, and αSyn under the standardized experimental conditions (Additional file 1: Figure S1). For Tau and αSyn, Western blots prepared from 10-day old adult fly heads and quantified relative to recombinant purified protein standards at known concentration (See Materials and Methods and Additional file 1: Figure S1). Aß was quantified using an established ELISA. Based on this, we estimate the relative molar concentration for expression of Aß, Tau, and αSyn driven by Rh1-Gal4 at 1:36:232, respectively. Animals were examined using complementary structural and functional assays to assess the toxic effects of each human protein in the fly retina. For structural studies, we used hematoxylin and eosin stained histologic sections to reveal photoreceptor integrity in the retina and lamina. Retinal function was probed using the ERG, permitting assessment of light-induced photoreceptor field potentials and efficacy of synaptic transmission [60].

### Tau

In prior work, expression of human Tau transgenes in the fly nervous system has been associated with substantial neurotoxicity, including in the fly eye, and these models have proven useful for both mechanistic investigation and genetic screening [19]. The most widely deployed assay has been the “rough eye”, typically induced using the *GMR-GAL4* driver, in which disrupted retinal development leads to a reduced eye size and a roughened surface. While the Tau-induced rough eye has shown great utility for genetic studies, especially modifier screens [1, 5, 52], potential limitations include the developmental component, as well as an inability to examine age-dependent changes, since the phenotype is established during development. Previously published and well-characterized *UAS:Tau* transgenic lines [62], expressing the 383 amino acid, wild-type human 0N4R isoform, were crossed to the *Rh1* driver. Compared to the rough eye caused by other eye drivers, the external eye appearance of *Rh1 > Tau* is indistinguishable from control animals (Additional file 2: Figure S2).

ERGs permit assessment of light induced field potentials serving as a sensitive indicator of photoreceptor function, retinal integrity/degeneration, and efficacy of synaptic transmission [60]. On eclosion (1 day old), *Rh1 > Tau* flies demonstrated normal ERGs, consisting of an approximately 10 mV, light induced depolarization (Fig. 1). However, with aging, Tau expression was associated with a significant loss of the light-evoked ERG response, consistent with progressive photoreceptor dysfunction. By contrast with preserved ERGs in aged *Rh1-GAL4* flies, *Rh1 > Tau* showed a 51 % reduction (*p* < 0.001) in phototransduction (depolarization amplitude) by 10 days progressing to an 81 % loss (*p* < 0.001) by 30 days (Fig. 1). We also quantified changes in the ERG ON/OFF transient responses, which denote synaptic neurotransmission. Under the conditions used for ERG recordings, it was difficult to detect robust reductions in the ON transient potential due to its small magnitude (~0.5 mV in *Rh1-GAL4 / +* controls). However, at all three time points examined, we observed significant reductions in the ERG OFF transient potential compared to controls (Fig. 1d).

**Fig. 2:**
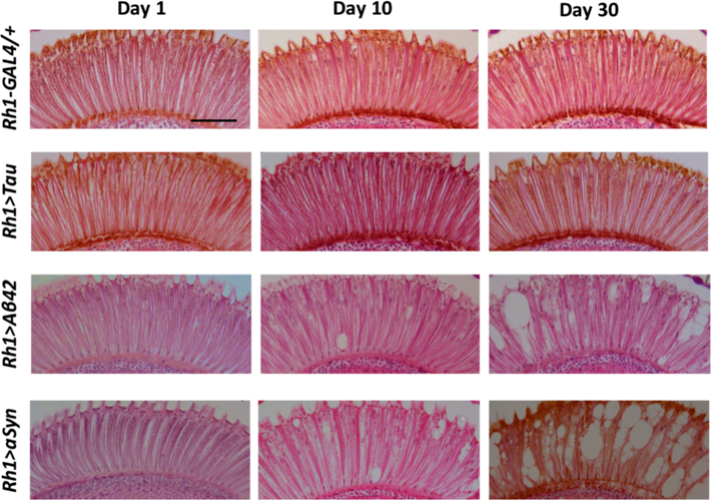
Representative frontal sections of the *Drosophila* retina stained with hematoxylin and eosin demonstrate age-dependent vacuolar change and tissue loss in *Rh1 > Aß* and *Rh1 > αSyn*, but relative preservation *Rh1 > Tau*. Scale bar: 50 μm

**Fig. 3:**
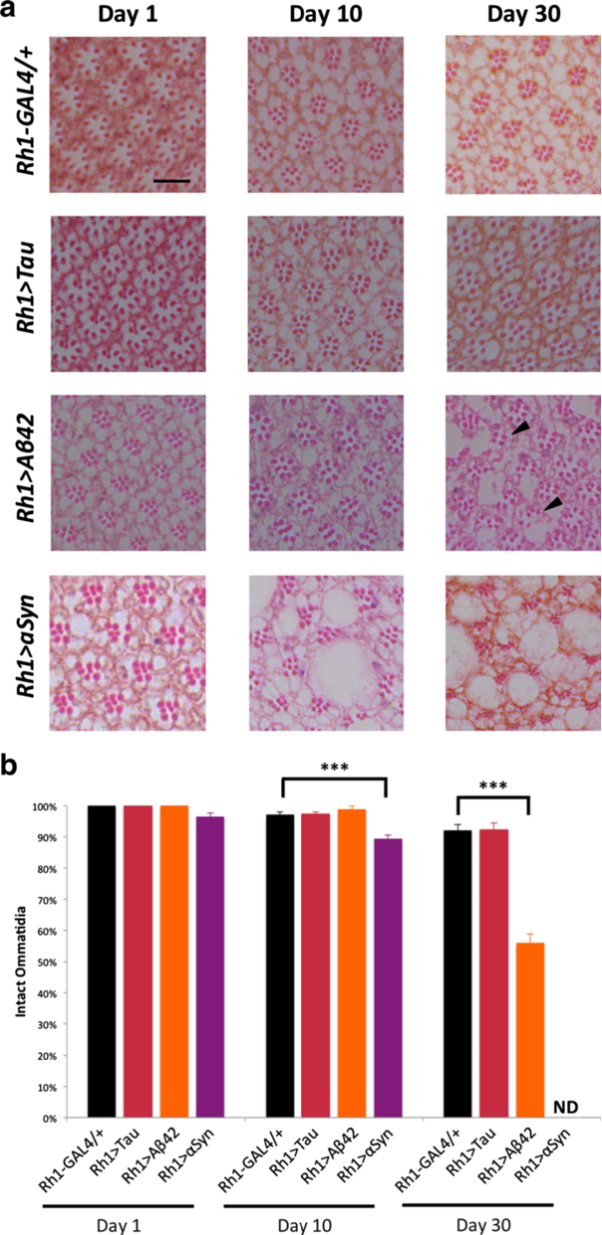
**a** Representative tangential sections of *Drosophila* retina stained with hematoxylin and eosin show progressive neuronal loss in *Rh1 > Aß* and *Rh1 > αSyn*, but not in *Rh1 > Tau*. Arrowheads highlight representative ommatidia with < 7 rhabdomeres. Scale bar: 10 μm. See also Additional file 3: Figure S3 for low-power views. **b** Photoreceptor loss in tangential sections was quantified based on the percentage of ommatidia with a preserved complement of 7 rhabdomeres at each timepoint (2-way ANOVA model F-test, *p* < 0.001). In 30 day-old animals, only 56 % of ommatidia in retinae from *Rh1 > Aß* flies had the full complement of 7 rhabdomeres, compared with 92 % in *Rh1-GAL4/+* controls (*p* < 0.001), whereas no significant change was seen in *Rh1 > Tau* (92 %). Given the severity of tissue loss at 30 days, it was not possible to identify preserved ommatidia in *Rh1 > αSyn* flies (ND); however, in 10 day-old animals, 89 % of ommatidia were intact, compared to 97 % of controls (*p* < 0.001), consistent with photoreceptor loss. Approximately 100 ommatidia were examined per 100x field of view for at least 4 animals per genotype. Total number of retinae examined (genotype, n_1_, n_10_, n_30_): *Rh1-Gal4/+*, 9, 8, 7; *Rh1 > Tau*, 7, 7, 10; *Rh1 > Aß*, 7, 8, 8; *Rh1 > αSyn* 7, 7, ND. Subsetted t-tests were performed for post-hoc comparisons of control animals with each experimental genotype, considering each timepoint independently, and p-values were adjusted using the Bonferroni method. At Day 1, SEM = 0 for *Rh1-Gal4/+*, *Rh1 > Tau*, and *Rh1 > Aß*. ***, *p* < 0.001

In order to assess the integrity of the *Drosophila* visual system, and to examine for photoreceptor cell loss, we prepared semi-thin sections of fixed and paraffin-embedded heads, staining with hematoxylin and eosin. Frontal (Fig. 2) and tangential (Fig. 3 and Additional file 3: Figure S3) microtome sections provide complementary views of retinal structure, allowing detection of any vacuolar changes and tissue architectural disruption. Tangential sections through photoreceptors readily permit sensitive detection of photoreceptor cell loss through counts of rhabdomeres, specialized organelles that mediate phototransduction. In contrast to the rapidly progressive loss of ERG responses, we did not observe any significant histologic changes in the retina in *Rh1 > Tau* flies; numbers and morphology of photoreceptors were preserved at all timepoints (Figs. 2 & 3). At 30 days, 92 % of ommatidia in quantified sections from *Rh1 > Tau* retinae demonstrated intact ommatidia with 7 visible rhabdomeres at the level examined, which was not significantly different from *Rh1-GAL4/+* controls (Fig. 3). Thus, when expressed in adult photoreceptors, human Tau induces progressive neuronal dysfunction, independent of any detectable neuronal loss or obvious retinal tissue changes.

### Aß42

Similar to Tau, Aß expression has previously been demonstrated to be toxic to *Drosophila* eye tissue, causing both disrupted architecture and a rough eye phenotype [10, 21]. These studies have also predominantly relied on the *GMR-GAL4* expression driver, which expresses throughout development, including both photoreceptors and supporting, non-neuronal cells, and produces a rough eye. When expression of the 42-amino acid Aß peptide (Aß42, referred to hereafter as Aß) is restricted to adult, differentiated photoreceptors, using *Rh1 > Aß*, animals have wildtype appearing eyes upon visual inspection, without a detectable roughening (Additional file 2: Figure S2). In contrast to *Rh1 > Tau*, ERG recordings from *Rh1 > Aß* animals do not show any evidence of rapidly progressive loss in neuronal function (Fig. 1). In fact, both photoreceptor depolarization and the associated transient responses were significantly increased in *Rh1 > Aß* flies at multiple timepoints. On examination of retina histology, 1-day old animals showed normal appearing retinal tissue morphology and architecture (Fig. 2), and a full complement of photoreceptors was observed in each ommatidium on tangential sections (Fig. 3). Upon aging, however, we observed progressive vacuolar changes affecting the retina, similar to those previously reported in *GMR > Aß* flies [21]. In addition, by 30 days we detected significant reductions in rhabdomere numbers (*p* < 0.001) consistent with neuronal loss, such that only 57 % of ommatidia in retinae from *Rh1 > Aß* flies were intact, whereas similar changes were not seen in *Rh1 > Tau* (Fig. 3b). Thus, compared with Tau, Aß induces a markedly distinct pattern of retinal neurodegeneration, consisting of progressive structural degeneration and neuronal loss with overall preserved photoreceptor functioning.

### αSyn

Unlike Aß and Tau, αSyn expression in the *Drosophila* eye using the *GMR-GAL4* driver line does not produce a rough-eye phenotype. However, modest evidence of toxicity has been reported on retinal histology [2, 18], including vacuolar changes, and one study reported ERG changes potentially consistent with disrupted neurotransmission [25]. When previously published *UAS:αSyn* lines [18] were expressed using the *Rh1* driver, we did not detect any appreciable changes in the ERG depolarization, including with aging, and only minimal changes were detected on retinal histology (Additional file 4: Figure S4). Based on the discovery of rare familial forms of synucleinopathy caused by *SNCA* locus multiplication [53], αSyn neurotoxicity is dose-sensitive, and this conclusion has also been supported by studies of mammalian models [16]. We therefore sought to develop and test new αSyn transgenic lines, in which codon usage in the full-length human αSyn cDNA was optimized for *Drosophila*, permitting more efficient protein translation. Indeed, based on western blot analysis, these lines demonstrate significantly increased αSyn expression levels (Additional file 4: Figure S4). Consistent with this observation and compared to αSyn transgenic flies without codon-optimization, files expressing codon-optimized αSyn under control of the *Rh1-GAL4* driver (hereafter referred to as *Rh1 > αSyn*) demonstrated a markedly enhanced toxicity profile. Using the ERG assay, we find that depolarization amplitude is initially preserved, but exhibits a modest but significant, age-dependent decline (versus *Rh1 / +* controls) which is apparent at 30 days (8 % reduction, p < 0.05) (Fig. 1b). ERG off transient potentials show a consistent, age-dependent loss in *Rh1 > αSyn* flies (Fig. 1d). On histological analysis, the retinae of *Rh1 > αSyn* flies initially display no obvious morphological defects, but aging leads to progressive vacuolar changes (Fig. 2) and rhabdomere loss (Fig. 3), ultimately leading to substantial architectural disruption and tissue destruction. In 10 day-old animals, we detected modest but significant reductions in rhabdomere numbers (p < 0.001) consistent with neuronal loss, such that 89 % of ommatidia in retinae from *Rh1 > αSyn* flies were intact, compared to 97 % in controls (Fig. 3b). By 30 days, the severity of tissue loss and architectural distortion made it impossible to identify preserved ommatidia. In sum, *αSyn* expression shows a third characteristic profile of neurodegeneration in the *Drosophila* visual system distinct from either Tau or Aß, causing substantial age-dependent structural degenerative changes, including cell death, with comparatively delayed, modest functional deficit based on ERG depolarization.

### Transmission electron microscopy

**Fig. 4:**
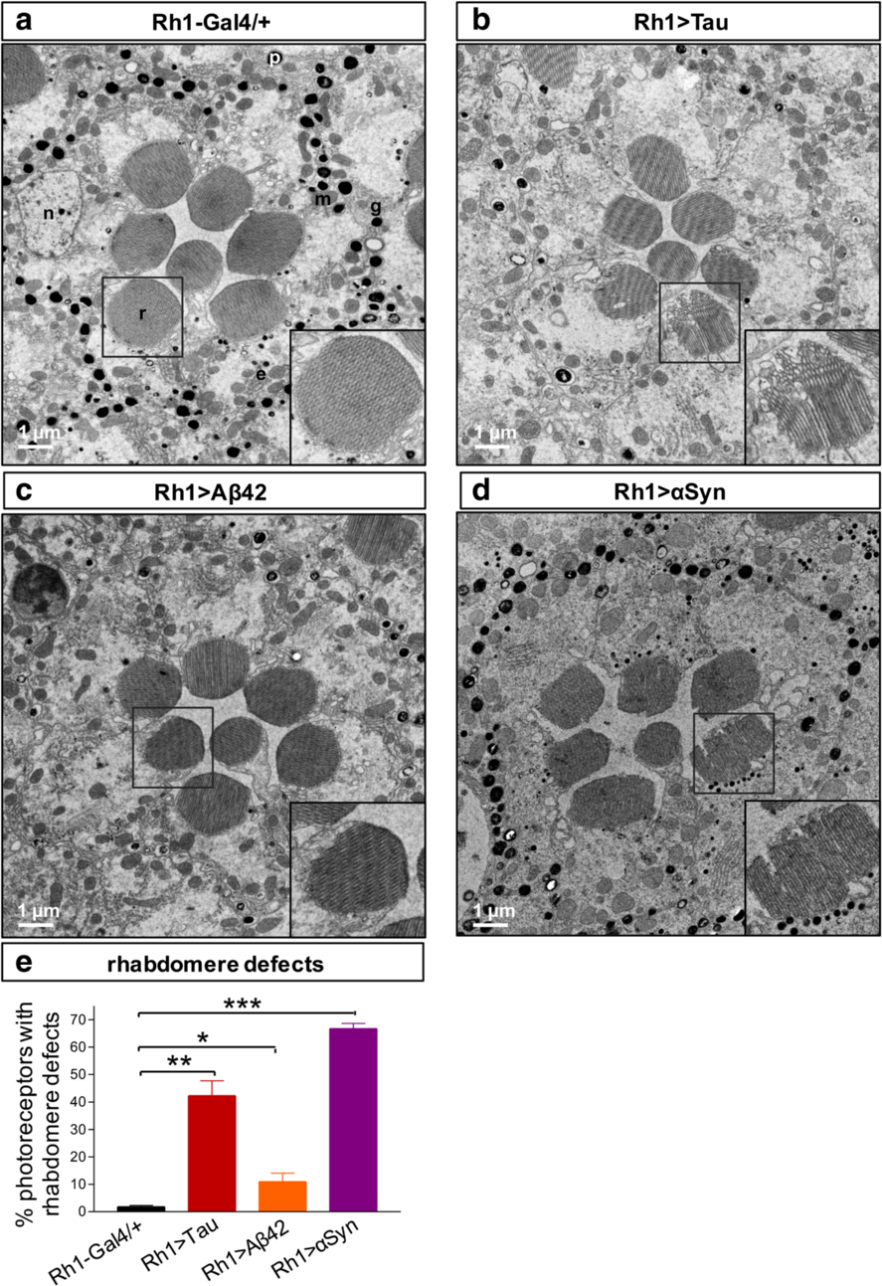
Compared to controls (**a**, *Rh1-GAL4 / +*), TEM reveals modest ultrastructural defects in rhabdomeres of *Rh1 > Tau* **b**, *Rh1 > Aß* **c** and *Rh1 > αSyn* **d** flies at 10 days. **a** Photoreceptor subcellular organelles are labelled, including nucleus (n), rhabdomere (r), mitochondria (m), and rough endoplasmic reticulum (e). Non-neuronal pigment (p) cells and glia (g) are also noted. **e** Frequency of photoreceptors with rhabdomere structural defects was quantified from electron micrographs taken from 3 distinct animals per genotype. Total number photoreceptors quantified (genotype, n): *Rh1-GAL4/+*, 614; *Rh1 > Aß*, 326; *Rh1 > Tau*, 389; and *Rh1 > αSyn*, 330. Scale bar: 1 μm. Statistical comparisons were implemented using a two-tailed student’s t-test. *, *p* < 0.05; **, *p* < 0.01; ***, *p* < 0.001

**Fig. 5:**
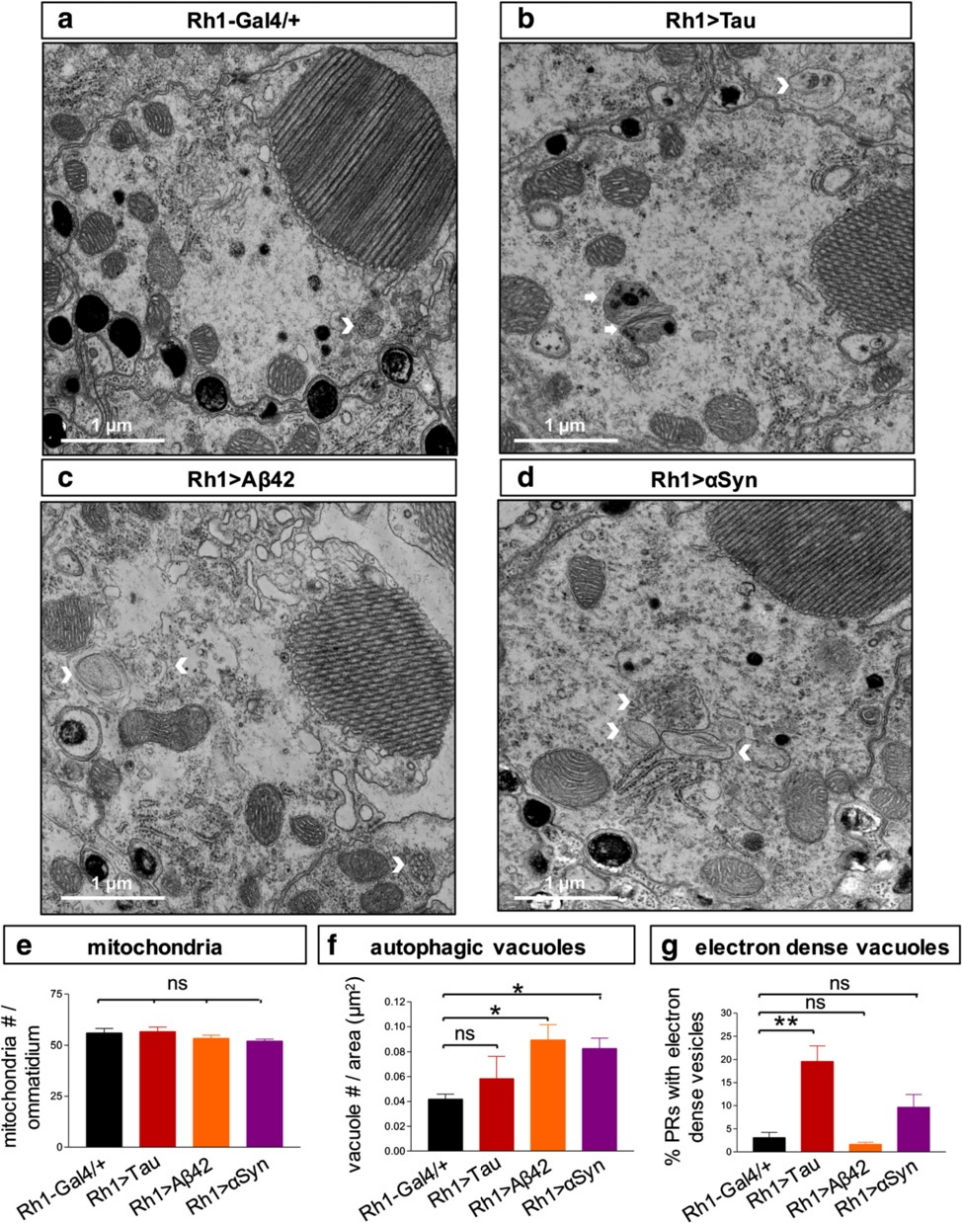
Compared to controls (**a**, *Rh1-GAL4 / +*), TEM reveals evidence of altered autophagy and endo-lysososomal flux in *Rh1 > Tau* **b**, *Rh1 > Aß* **c** and *Rh1 > αSyn* **d** flies at 10 days, but unchanged mitochondria. Autophagic vacuoles (arrowheads) and electron dense vacuoles (arrows) are indicated. Quantification **e**-**g** of ultrastructural features was based on electron micrographs from 3 distinct animals per genotype. **e** Number of mitochondria were quantified per ommatidium. Total number ommatidia counted (genotype, n): *Rh1-Gal4/+*, 18; *Rh1 > Aß*, 21; *Rh1 > Tau*, 20; and *Rh1 > αSyn*, 18. **f** Aß and αSyn induce autophagic vacuole formation in the *Drosophila* retina. Distinct vacuolar compartments representing autophagic flux were quantified per photoreceptor area (n / μm^2^), including autophagosomes, multi-vesicular bodies, multi-lamellar bodies, and lysosomes. **g** Increased frequency of electron dense vacuoles following expression of Tau, potentially consistent with the appearance of telolysosomes (also called lipofuscin granules). Total number photoreceptors quantified (genotype, n): *Rh1-GAL4/+*, 340; *Rh1 > Aß*, 269; *Rh1 > Tau*, 320; and *Rh1 > αSyn*, 257. Scale bar: 1 μm. All statistical comparisons were implemented using a two-tailed student’s t-test. *, *p* < 0.05; **, *p* < 0.01

In order to reveal potential mechanisms underlying the distinct patterns of toxicity seen using medium throughput assays, and to understand the discordance between structural and functional assays, we performed TEM to examine photoreceptor ultrastructure following expression of Aß, Tau, or αSyn with the *Rh1-GAL4* driver. We used identical conditions to those described above, and performed our analysis at 10 days in order to capture the evolution of age-dependent changes. Based on retinal TEM, all three toxic proteins induced modest disruptions in photoreceptor rhabdomeres (Fig. 4), specialized membrane-rich, electron dense organelles that mediate phototransduction and thereby generate the ERG depolarization. Compared to *Rh1 / +* controls, expression of Tau, Aß, or αSyn caused subtle defects in rhabdomere architecture, including apparent splitting / “fraying” at the edges, vacuolar changes, and other irregularities. However, the frequency of these changes did not appear to correlate well with the extent and timing of functional deficits as revealed by ERG. Consistent with findings on retinal histology, rhabdomere disruption was most frequent in *Rh1 > αSyn* (~65 %, Fig. 4e) even though significant changes in ERG depolarization are not observed at this timepoint. Conversely, despite the profound reduction in the ERG response characteristic of *Rh1 > Tau* flies at 10 days (Fig. 1), the majority of photoreceptors had preserved rhabdomeres. The resolution afforded by TEM also allowed us to examine several subcellular systems implicated in neurodegeneration (Fig. 5). While mitochondrial numbers and morphology were preserved (Fig. 5e), we observed evidence of increased endolysosomal-dependent processes (Fig. 5f & g). Specifically, in both *Rh1 > Aß* and *Rh1 > αSyn*, we see increased numbers of multivesicular or multilamellar bodies, consistent with altered autophagic flux, whereas in *Rh1 > Tau,* a preponderance of electron dense vesicles were seen consistent with the appearance of telolysosomes (also called residual bodies or lipofuscin granules). Electron-dense material, possibly consistent with granular tau oligomeric aggregates, has previously been observed in Tau transgenic flies [15].

**Fig. 6:**
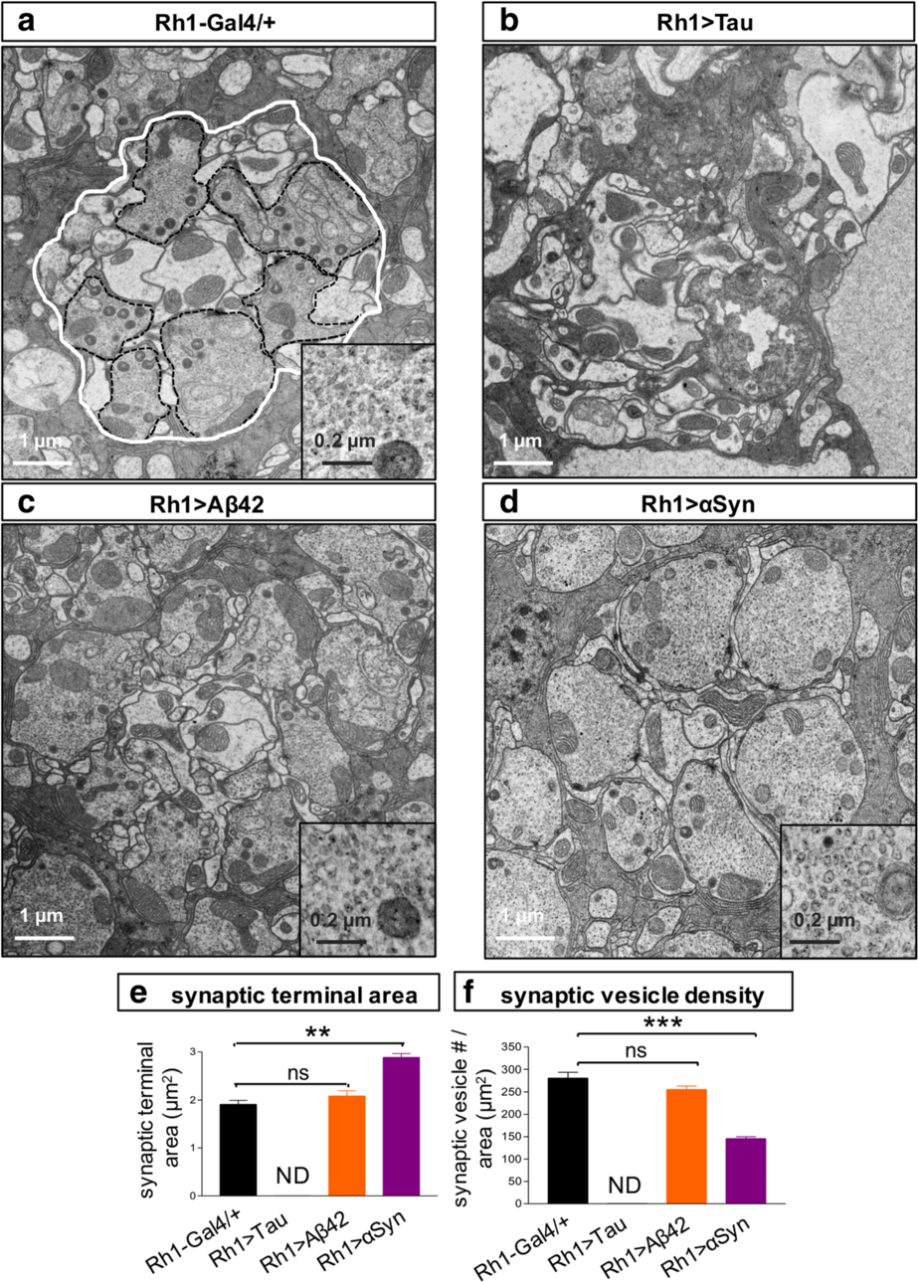
TEM of the *Drosophila* lamina (**a**, *Rh1-GAL4 / +*) highlights photoreceptor synaptic terminals (dashed black outline) arrayed into cartridges (white outline). Ultrastructural evidence of synaptotoxic mechanisms is seen in *Rh1 > Tau* **b**, and *Rh1 > αSyn* **d** flies at 10 days, but not in *Rh1 > Aß* **c**. Insets show higher power views of synaptic terminal contents, revealing apparent enlargement and heterogeneous morphology of synaptic vesicles in *Rh1 > αSyn* (compare panel **d** inset with **a** and **c**). **e**-**f** Quantification of ultrastructural features based on electromicrographs from 3 distinct animals per genotype. Due to architectural disorganization and altered morphologic appearance, it was not possible to quantify synaptic terminal features in *Rh1 > Tau* animals. **e** αSyn induces an enlargement in synaptic terminal area. Total number synaptic terminals quantified (genotype, n): *Rh1-GAL4/+*, 201; *Rh1 > Aß*, 182; and *Rh1 > αSyn*, 185. **f** Decreased synaptic vesicle density following αSyn expression. Synaptic vesicles were quantified per photoreceptor terminal area (n / μm^2^). Scale bars: 1 μm (main panel), 0.2 μm (inset). All statistical comparisons were implemented using a two-tailed student’s t-test. **, *p* < 0.01; ***p < 0.001

We next examined the organization and morphology of photoreceptor synaptic terminals, based on TEM of the lamina. In control animals (*Rh1 / +*), TEM reveals a regular array of “cartridges” in which 6 photoreceptor synaptic terminals converge and surround the centrally located dendritic projections from second order neurons in the *Drosophila* visual system (Fig. 6a). The cytoplasm of the photoreceptor terminals appears electron dense relative to the dendritic processes due to the synaptic vesicular contents. Expression of Tau or αSyn, but not Aß, significantly disrupted photoreceptor synaptic terminal organization and/or morphology. Remarkably, in *Rh1 > Tau* animals examined at 10 days, it was no longer possible to discriminate photoreceptor terminals (Fig. 6a), perhaps due to a paucity of synaptic vesicles, and the overall architecture of the lamina was disturbed. By contrast, in *Rh1 > αSyn*, overall lamina organization was preserved; however, synaptic terminals were significantly enlarged, assuming a bulbous appearance (Fig. 6d and e), and synaptic vesicle density was found to be decreased (Fig. 6f). Additionally, αSyn expression appeared to alter synaptic vesicle size, leading to enlarged and more heterogeneous vesicles (inset, Fig. 6d). In sum, TEM reveals significant but distinct patterns of synaptotoxicity following expression of Tau and αSyn in adult photoreceptors.

## Discussion

We report on parallel, systematic assessments of both structural and functional changes occurring in the *Drosophila* retina following expression of human Tau, Aß, and αSyn under identical conditions. Remarkably, these toxic protein species are discovered to recapitulate distinct profiles of neurodegeneration, and the findings highlight a surprising “uncoupling” between neuronal dysfunction and retinal destruction/cell loss. Expression of Tau in the adult retina causes a significant and progressive decline in photoreceptor function, based on the ERG, whereas tissue histology reveals preserved retinal architecture without evidence of structural degeneration. As discussed further below, Tau-related neuronal dysfunction has been reported in several prior *Drosophila* studies using a variety of complementary expression conditions and assays [12, 36, 38]. By contrast, we show that Aß expression has minimal impact on the ERG but causes progressive retinal vacuolar changes and associated rhabdomere loss. αSyn expression—based on new, codon-optimized transgenes—generates a third profile consisting of rapid, age-dependent histologic destruction of retinal tissue with comparatively late, and modest change in photoreceptor field potentials. Finally, ultrastructural analyses reveal evidence of altered endolysosomal pathways following expression of all three proteins, and in the case of Tau and αSyn, synaptotoxic mechanisms are strongly implicated. Interestingly, mitochondrial numbers and morphology were preserved in all 3 models. The assays employed in our studies (histology, ERG, and TEM) are highly complementary, allowing assessments of neurodegenerative changes at the macro-, micro-, and ultra-structural levels as well as correlation with retinal neurophysiology. Based on numerous prior studies, the mechanisms of Aß-, Tau-, and αSyn-triggered neuronal injury in AD and PD, respectively, have many features in common, including aggregation, pathologic propagation [59], and overlapping cytotoxic profiles (e.g. synaptic changes [4, 56], mitochondrial dysfunction/oxidative stress [24, 61], and altered proteostasis [34]). Despite these parallels, the distinct degenerative phenotypes observed following expression of Tau, Aß, and αSyn expression in the *Drosophila* eye suggest these protein species engage at least partially non-overlapping downstream effector mechanisms.

While we have taken several steps to standardize expression conditions for Tau, Aß, and αSyn, it is not possible to exclude the contribution of variation in transgene/protein expression levels to the observed toxicity profiles. Indeed, the neurotoxicity of these protein species is highly dose-dependent in most experimental systems, and human genetic variants that increase Aß and αSyn production are risk factors for AD and PD, respectively [32, 46]. Based on quantitative Western blots and ELISA, we estimated the relative expression of Aß, Tau, and αSyn under our experimental conditions was approximately 1:30:200 (by molarity), respectively (Additional file 1: Figure S1). The comparatively high αSyn expression levels likely relates to codon-optimization, which achieves ~20-fold higher expression compared to previously published transgenic strains (Additional file 4: Figure S4). While we estimate that non-codon optimized αSyn has more comparable expression levels (Aß:Tau:αSyn = 1:30:10), this line demonstrates minimal toxicity in our assays, perhaps consistent with more robust neuronal compensatory mechanisms. Nevertheless, variation in protein expression levels seem unlikely to account for many of our observations, since higher expression was in fact a poor predictor of enhanced toxicity. For example, despite its relatively *higher* expression over Tau, αSyn caused a comparatively modest and late disruption in the ERG. Moreover, despite the significantly *lower* expression, Aß but not Tau triggered retinal vacuolar changes and rhabdomere loss. In the future, it may be informative to repeat our studies following codon-optimization of Aß42 and Tau and/or with transgenic strains that use the same genomic insertion site.

One strength of our study is the choice of expression conditions facilitating comparison of toxicity profiles for Tau, Aß, and αSyn in adult neurons. We selected the *Rh1-GAL4* expression driver which is activated shortly prior to eclosion of adult flies within mature, fully differentiated photoreceptors and following patterning and morphogenesis of the eye [37, 47, 48, 51]. While the spatiotemporal expression profile of the *Rh1* promoter has been extensively-characterized, we cannot exclude the possibility of low-level “leaky” transgene expression. In order to further suppress expression during development, including late pupal stages, crosses were established at 18 °C and adult animals were shifted to 25 °C following eclosion. Most prior work using retinal phenotypes for fly neurodegenerative models have relied on drivers that express during eye development (*GMR-, gl-,* and *Sev-GAL4*). When Aß [21] or Tau [62] is expressed in this manner, retinal morphogenesis is disrupted resulting in a “rough eye”. Importantly, the rough eye phenotype is readily amenable to enhancer/suppressor screening, and successful large-scale and unbiased modifier studies have been performed in both Tau [1, 5, 52] and Aß [9] transgenic flies. However, these developmental driver lines make it difficult to study the toxicity of Tau in mature neurons, potentially most relevant for adult-onset neurodegenerative disorders such as AD and PD. Notably, the ERG assay used here has also been deployed successfully for large-scale genetic screens [64]. Another potential advantage of *Rh1* is its selectivity for retinal photoreceptors compared to *GMR-GAL4,* which is expressed more broadly within both neuronal and supporting cells, including glia. Based on studies in the brain, Tau can also recapitulate toxicity in *Drosophila* glia, and neuron-glia interactions can influence overall levels of degeneration in the nervous system [14]. In sum, in contrast to much prior work using the *Drosophila* eye as a tissue for the study of neurodegeneration, our findings selectively highlight the impact of Aß, Tau, or αSyn in post-mitotic neurons.

One unexpected finding is the progressive photoreceptor dysfunction in *Rh1 > Tau* flies that occurs in the absence of apparent retinal histologic degeneration or neuronal death. This contrasts not only with the effects of Aß or αSyn when expressed under identical conditions, but also with the profound rough eye and associated retinal histologic changes following developmental expression of Tau (e.g. *GMR-GAL4*). Even with the increased resolution provided by TEM, retinal architecture was surprisingly preserved with significant loss of photoreceptor depolarization out of proportion to changes in the responsible cellular compartment/structures (i.e. ommatidia/rhabdomeres). Notably, prior studies of Tau-induced progressive neurodegeneration in the adult brain [62] have relied on drivers, such as *elav-GAL4*, which are also expressed early in neuronal differentiation. Overall brain morphology is preserved in these flies; although aberrant development of selected neuronal structures, such as the mushroom bodies, have been documented [30]. Potentially related to our observations of “uncoupling” between neuronal dysfunction and degeneration, Kosmidis et al. found that certain mutant forms of Tau expressed under the control of *elav-GAL4* disrupt *Drosophila* olfactory memory, while apparently preserving mushroom body morphology [30]. In the context of glia, Colodner and Feany demonstrated that conditional expression of wild-type Tau in the adult fly brain is capable of inducing progressive apoptotic degeneration [14]. Under conditions in which neuronal expression of Tau is restricted to the adult brain, Papanikolopoulou and Skoulakis showed that flies have decreased survival, but neuronal loss and/or vacuolar changes was not examined [45]. In studies of the larval peripheral nervous system, Mudher et al. found that Tau is capable of disrupting axonal transport [38], and Chee et al. documented Tau-induced synaptic dysfunction [12], both in the absence of apparent neuronal death. Our findings of preserved photoreceptors in the retinae of *Rh1 > Tau* flies appears contradictory to recent findings by Gorsky et al. [22]; however, our studies differ in the specific Tau isoform examined, expression driver/conditions, and assays employed. Tau phosphorylation is also likely an important determinant of the specific profile of Tau toxicity, based on numerous studies in *Drosophila* [1, 11, 42, 45, 57] and many other experimental systems [23]. In sum, the alternative outcomes of neuronal dysfunction versus death following Tau expression may be sensitive to many factors, including timing, cell-type, protein levels, isoform, and post-translational modifications.

Early synaptotoxic mechanisms are emerging as important in the pathogenesis of both AD and PD [44, 49, 56]. Based on TEM, we observed profound disruption of photoreceptor terminals in both *Rh1 > Tau* and *Rh1 > αSyn* flies; although, the character of the specific defect was distinct. Tau was associated with a disorganized lamina architecture, and it was no longer possible to differentiate photoreceptor terminals. This is consistent with prior findings of reduced synaptic markers in Tau flies out of proportion to neuronal cell loss [35]. One attractive hypothesis is that these changes might arise from Tau-mediated disruption of axonal transport, leading to impaired delivery of mitochondria and synaptic vesicles and resulting in terminal collapse, consistent with prior observations in a variety of Tauopathy models [17, 26, 38]. Following *αSyn* expression, TEM revealed an expansion of the synaptic terminals and enlarged but decreased density of synaptic vesicles. Notably, similar changes have been documented in the brains of αSyn transgenic mice [6, 41, 50]. αSyn avidly binds cellular membranes, including synaptic vesicles and other endomembrane organelles, likely accounting for its conserved activity to remodel and disrupt such systems[4]. Consistent with our TEM findings of disrupted synaptic ultrastructure, Tau and αSyn were each additionally associated with progressive decline in the ERG OFF transient potentials, suggesting impaired neurotransmission [60]. Despite the early and potent synaptotoxicity associated with Aß in many other systems [44, 56], we did not detect significant disruptions in the lamina ultrastructure of *Rh1 > Aß* flies. It will be important in the future to examine for possible emergence of synaptic or other ultrastructural changes at later timepoints since the Aß-induced histologic findings are quite sparse at 10 days and could potentially be missed by TEM due to sampling. Interestingly, despite the age-dependent photoreceptor loss triggered by Aß, we observed an initial increase in both the ERG ON/OFF transients along with the overall increased depolarization amplitude. One speculative possibility is that these findings reflect cellular and synaptic functional compensatory mechanisms. While additional studies will be required to understand these dynamic neurophysiologic changes, we note that apparently paradoxical effects have been previously reported for Aß species at *Drosophila* neuromuscular junctions and in mammalian neurons [13, 65].

## Conclusions

Based on a combination of functional neuroimaging and clinicopathologic correlation studies, there is increasing recognition that synaptic and circuit dysfunction contribute significantly to AD and PD manifestations, and may in fact precede neuronal death and tissue atrophy [27, 29]. It will be important to directly address this experimentally in model systems, and to develop assays that allow mechanistic studies of functional degenerative changes. In *Drosophila,* Aß, Tau, and αSyn transgenic models have been previously utilized for electrophysiological studies at the larval neuromuscular junction [8, 12, 13], but this approach is potentially limited for the analysis of age-dependent progressive changes in adult neurons. Nervous system function has also been probed in fly disease models based on learning and locomotion in adult animals [19]; however, as discussed above, the relative contribution of neuronal loss versus dysfunction to these behaviors remains to be carefully elucidated. In sum, the parallel studies of retinal ERG, histology, and TEM reported here provide a foundation for future genetic dissection of mechanisms underlying progressive synaptic and functional disruption in transgenic models of AD and PD, and its relation to the cumulative and potentially irreversible burden from tissue destruction and neuronal loss.

## Additional files

Additional file 1: Figure S1. Quantitation of transgenic expression of Tau, Aβ, and αSyn. (PDF 130 kb) Additional file 2: Figure S2. External eye appearance of genotypes evaluated in this study. (PDF 177 kb) Additional file 3: Figure S3. Low power histologic sections. (PDF 158 kb) Additional file 4: Figure S4. Expression and toxicity of non-codon optimized αSyn. (PDF 141 kb)
