# Supplementary material for: Uncoupling neuronal death and dysfunction in Drosophila models of neurodegenerative disease
Source: Acta Neuropathol Commun. 2016 Jun 23;4:62. doi: 10.1186/s40478-016-0333-4 (PMC4918017; doi:10.1186/s40478-016-0333-4)
Supplement: Additional file 1: Figure S1. — Quantitation of transgenic expression of Tau, Aβ, and αSyn. (PDF 130 kb) [file 40478_2016_333_MOESM1_ESM.pdf]

Additional file 1: Figure S1. Quantitation of transgenic expression of Tau, Aß, and αSyn. Expression estimates for Tau (0.095 ± 0.01 μg) and αSyn (0.223 ± 0.02 μg) were determined on western blot relative to dilution of known recombinant, purified protein standards. Quantification of Aß (0.30 ± 0.02 ng) was based on established ELISA (Invitrogen, See Methods). Measurements were performed on heads from 10-day old adult animals (*Rh1>Tau, Aß,* or *αSyn*).

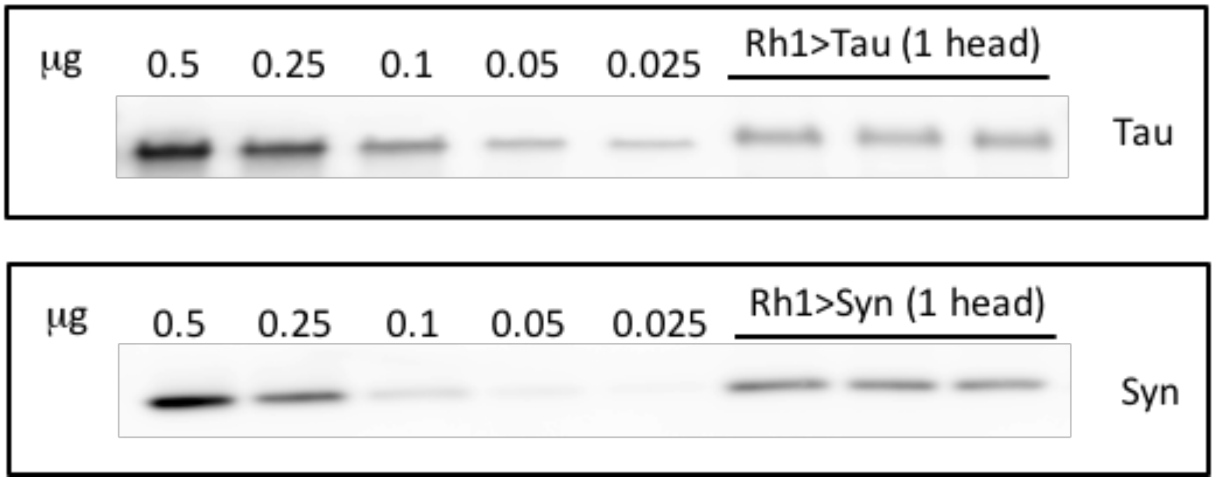

Rh1>	weight (μg)	MW (kDa)	relative molar concentration
**Aß**	0.0003	4.51	1
**Tau**	0.095	40	36
**αSyn**	0.223	14.46	232
