# Supplementary material for: Uncoupling neuronal death and dysfunction in Drosophila models of neurodegenerative disease
Source: Acta Neuropathol Commun. 2016 Jun 23;4:62. doi: 10.1186/s40478-016-0333-4 (PMC4918017; doi:10.1186/s40478-016-0333-4)
Supplement: Additional file 2: Figure S2. — External eye appearance of genotypes evaluated in this study. (PDF 177 kb) [file 40478_2016_333_MOESM2_ESM.pdf]

Additional file 2: Figure S2. Expression of human Tau, Aß, and αSyn using the *Rh1-GAL4* driver causes no external eye phenotypes. Variation in pigment results from varying dose of the *w+* marker gene.

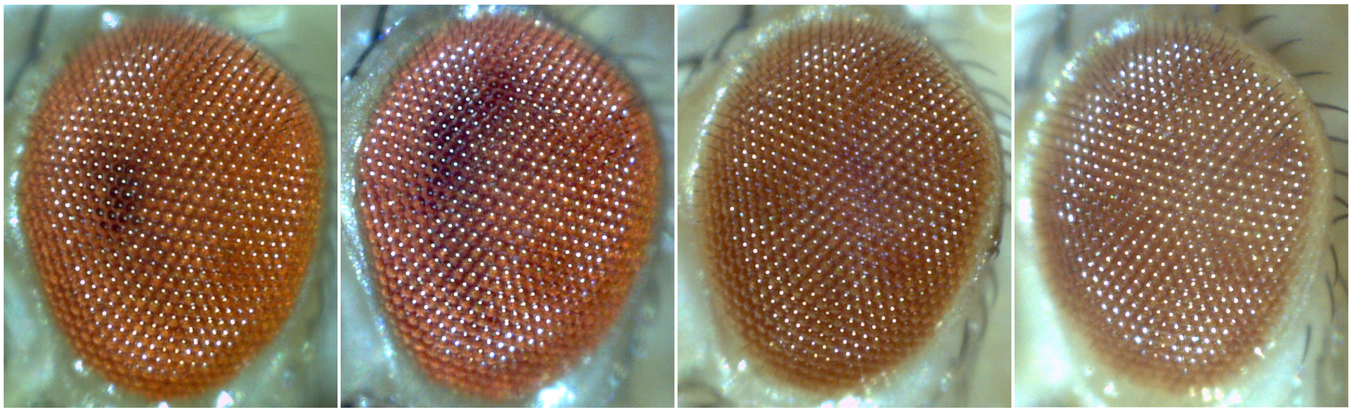

Rh1-GAL4/+ ***Rh1>Tau*** ***Rh1>Aß*** ***Rh1>αSyn***
