# Supplementary material for: Uncoupling neuronal death and dysfunction in Drosophila models of neurodegenerative disease
Source: Acta Neuropathol Commun. 2016 Jun 23;4:62. doi: 10.1186/s40478-016-0333-4 (PMC4918017; doi:10.1186/s40478-016-0333-4)

Additional file 3: Figure S3. For all sections displayed in Fig. 3, tangential sections are shown with expanded field of view. Scale bar: 20 μm.

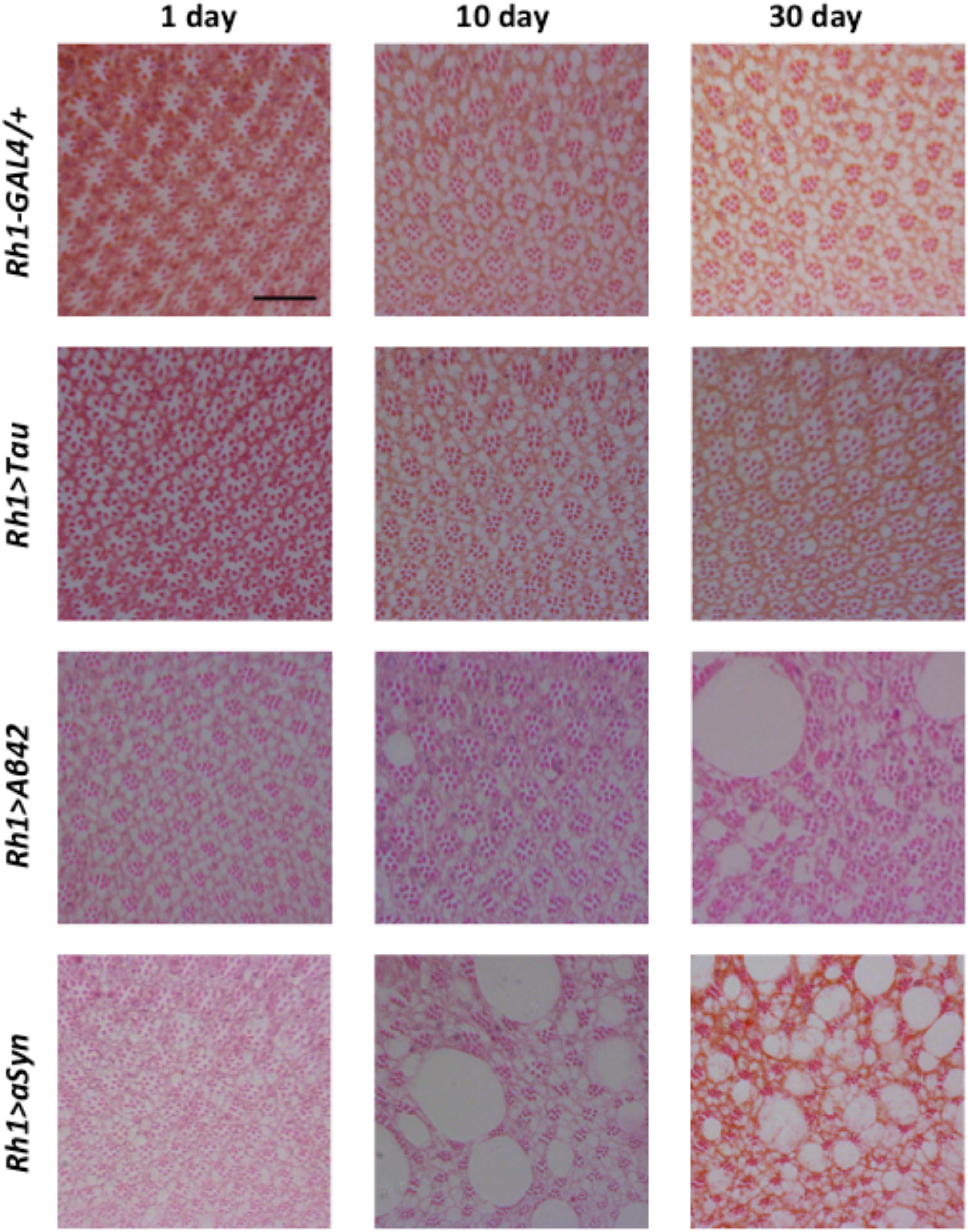

Supplement: Additional file 3: Figure S3. — Low power histologic sections. (PDF 158 kb) [file 40478_2016_333_MOESM3_ESM.pdf]
