# Supplementary material for: Uncoupling neuronal death and dysfunction in Drosophila models of neurodegenerative disease
Source: Acta Neuropathol Commun. 2016 Jun 23;4:62. doi: 10.1186/s40478-016-0333-4 (PMC4918017; doi:10.1186/s40478-016-0333-4)

Additional file 4: Figure S4. (TOP) Published, non-codon optimized (NCO) αSyn transgenic lines [ref. 18] show significantly reduced expression (~20-fold) compared to the codon-optimized (CO) transgene. (BOTTOM) *Rh1>αSyn*^{NCO} shows highly-attenuated retinal toxicity. ERG depolarization amplitude is preserved with aging. On retinal histology minimal vacuolar changes are observed (compare with Fig. 2 and Fig. 3); frontal and tangential sections are shown for 30 day old *Rh1>αSyn*^{NCO} animals).

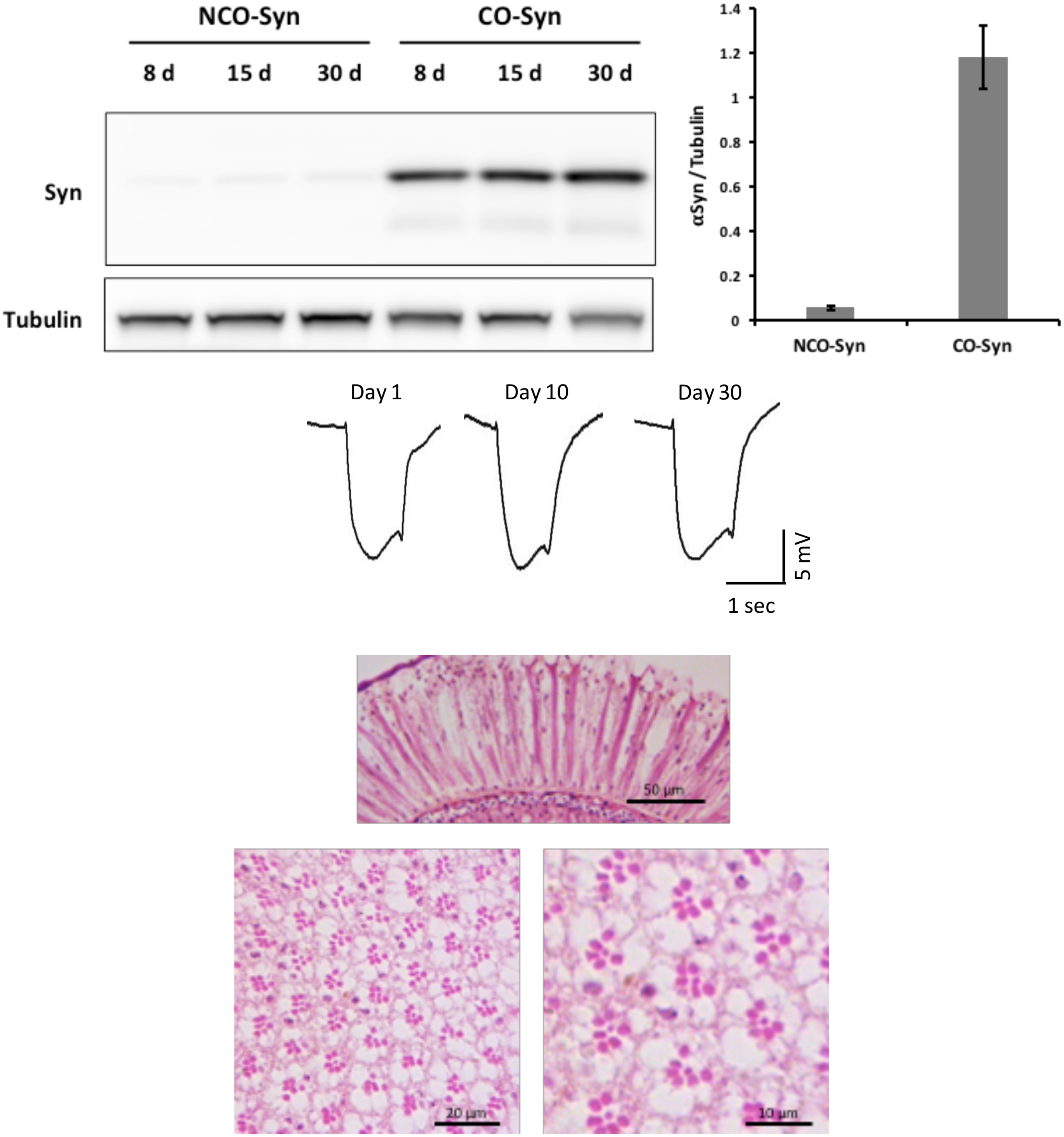

Supplement: Additional file 4: Figure S4. — Expression and toxicity of non-codon optimized αSyn. (PDF 141 kb) [file 40478_2016_333_MOESM4_ESM.pdf]
